# Recurrent Hodgkin's Disease Presenting as a Clinically Isolated Cavernous Sinus Syndrome

**DOI:** 10.1155/2021/3946231

**Published:** 2021-08-07

**Authors:** Aayesha Jalaluddin Soni, Edward Bernard Lee-Pan

**Affiliations:** Department of Neurology, University of Cape Town, Cape Town, South Africa

## Abstract

**Background:**

Hodgkin's disease involving the central nervous system is uncommon and is usually seen in patients with uncontrolled systemic disease or those who have had multiple episodes of recurrent disease. Common symptoms of intracranial Hodgkin's disease are motor and/or sensory deficits, headache, papilloedema, coma, and seizures. The rarity of Hodgkin's disease presenting with intracranial involvement is marked, but patients presenting with cavernous sinus syndrome is even rarer. Despite its rarity, the presence of a cavernous sinus syndrome in a patient with a known history of Hodgkin's disease warrants full utilization of modern diagnostic techniques in terms of investigation. *Case Presentation*. A 34-year-old woman, known with previous Hodgkin's lymphoma and now in remission for the past 7 years, presented with signs and symptoms suggestive of a left cavernous sinus syndrome. She was otherwise systemically well with no other complaints. Extensive investigations revealed no obvious cause for the cavernous sinus syndrome. A CT chest revealed subclinical axillary lymphadenopathy, which on excisional biopsy confirmed recurrent Hodgkin's disease. The patient's sole clinical presentation of her recurrent disease was the cavernous sinus syndrome, with no other clinically obvious systemic signs or symptoms to suggest a relapse. This was treated with steroids, and clinical improvement was noted; she was referred to oncology for extensive chemotherapy.

**Conclusions:**

Whilst there is a paucity of literature around this topic, it must be remembered by the clinician that cavernous sinus syndrome may be the sole clinical presentation of recurrent Hodgkin's disease. Even though it is reported that lymphomatous involvement in the brain usually occurs late in the disease process, this case is evidence that cavernous sinus syndrome may precede other systemic signs and symptoms. Recognising this possibility will ensure a rigorous search for Hodgkin's disease, an early and effective diagnosis, and a better prognosis in affected patients.

## 1. Background

Hodgkin's disease (HD) involving the central nervous system (CNS) is uncommon and is usually seen in patients with uncontrolled systemic disease or those who have had multiple episodes of recurrent disease. [1] There have been no identifiable risk factors for HD presenting with intracranial involvement. Whether at initial presentation or at relapse, the occurrence of intracranial HD without overt evidence of disease elsewhere is very rare. Whilst the literature acknowledges the rarity of HD presenting with CNS symptoms (especially in contrast to non-Hodgkin's disease which frequently presents in the CNS), patients presenting with cavernous sinus syndrome (CSS) is even rarer. There are only 2 other documented adult patients with HD in remission who presented with recurrence in the cavernous sinus [2] and no adult cases of primary HD presenting with isolated CSS. All of our knowledge on the topic is limited to isolated case reports in patients with salient features. Our patient presented clinically with a CSS only and no other systemic signs or symptoms to suggest a relapse of HD.

## 2. Case Presentation

A 34-year-old woman presented with an 8-day history of a progressively worsening left-sided headache, originating around her eye and radiating backwards. She also reported six days of worsening horizontal diplopia on binocular vision and more recent nausea. There were no other constitutional symptoms.

She has a ten pack-year smoking history, with otherwise sober social habits. On past medical history, she was previously treated for Hodgkin's lymphoma (HL), achieving remission in 2009. She was diagnosed with HL stage IIA in May 2007 and was treated with six cycles of ABVD chemotherapy, followed by DHAP chemotherapy and involved-field radiation therapy. In February 2009, she suffered a relapse of her HL which was treated with six cycles of OPEC chemotherapy. A PET scan after this treatment revealed no further active disease. There was no neurological involvement during either of these presentations. She then had yearly follow-up appointments with the oncology team until she was discharged in 2014 and considered to be in remission. Since then, she remained asymptomatic.

On examination, she was a healthy-looking young lady with no remarkable abnormalities noted on general, respiratory, or cardiovascular exam. Of note, there were no palpable lymph nodes globally. Neuro-ophthalmic examination revealed normal visual acuity and visual fields, and fundoscopy was normal, with no evidence of proptosis or chemosis. Neurological examination on admission revealed a left abducens nerve palsy and left-sided trigeminal sensory loss in the first and second divisions. The rest of the neurological exam was initially normal; however, within days of her admission, her symptoms progressed to involve the oculomotor nerve on the left as well. This was accompanied with worsening pain, consistent with involvement of the left cavernous sinus.

**Figure 1:**
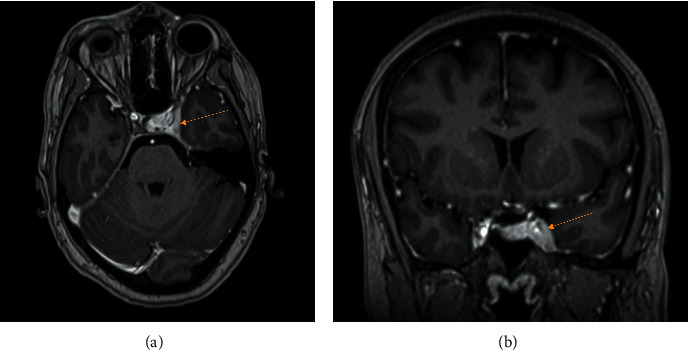
T1-weighted postcontrast MRI brain showing abnormal enhancement of the left cavernous sinus (arrows) in the axial and coronal views, respectively.

In terms of investigations, an extensive blood work-up revealed only a slightly raised ESR of 26 on admission. A full blood count, renal and liver function panel, thyroid function, beta D glucan, HIV and syphilis screen, hepatitis screen, and autoimmune screen were all normal and negative. A lumbar puncture revealed normal cerebrospinal fluid testing including cytology and flow cytometry. An MRI brain confirmed the presence of abnormal enhancement and expansion of the left cavernous sinus and paracavernous dura extending to the orbital apex and posteriorly to the tentorial insertion (Figure 1). The neurosurgical team was reluctant to perform a biopsy of the cavernous sinus before further systemic work-up had been performed. A CT of the neck, chest, abdomen, and pelvis revealed subclinical clustered right axillary lymphadenopathy (maximum diameter 15 mm) as the only finding. A mammogram noted the axillary lymph node complex, but was otherwise normal. Excisional biopsy of the right axillary lymph nodes revealed scattered Reed–Sternberg cells with prominent macronucleoli, with positive CD30 and CD15 noted on immunohistochemistry of the atypical cells.

The patient was diagnosed with recurrent Hodgkin's lymphoma (nodular sclerosing). She received intravenous steroids for the treatment of her left cavernous sinus syndrome which resolved clinically within five days and was referred to oncology for further management, which she is currently awaiting. A repeat MRI brain was not performed following clinical improvement.

## 3. Discussion and Conclusions

Cavernous sinus syndrome (CSS) is defined as ophthalmoplegia, chemosis, proptosis, Horner syndrome, or trigeminal sensory loss secondary to pathologic changes in or around the cavernous sinus [1]. Due to the fact that several cranial nerves including the oculomotor, trochlear, abducens, and the ophthalmic and maxillary parts of the trigeminal nerve can be wholly or partially involved in this syndrome, there is a range of clinical signs that may be elicited. Nevertheless, the diagnosis can be made clinically with reasonable confidence in the presence of a combination of deficits of these cranial nerves. There are many causes, principally due to infectious or noninfectious inflammation, vascular, traumatic, and neoplastic processes (Table 1) [1].

Retrospective and prospective cohort studies suggest that CNS involvement occurs in 0.2% to 0.5% of patients with systemic HD [3]. In a review of 2 large series which included 780 and 2185 patients, the incidence of intracranial involvement of HD was reported as only 0.4% and 0.5%, respectively [3,4]. The main sites of brain involvement were the parenchyma (64%) and dura (19%) [5]. The mechanism of brain metastasis is either direct tumour extension through the skull bone or systemic haematogenous dissemination, with the latter being the most common [6].

The most common CNS symptoms of intracranial HD are motor and/or sensory deficits, headache, papilloedema, coma, and seizures [7].

CNS lesions in HD pose a diagnostic dilemma in view of the rare occurrence of brain involvement by Hodgkin's disease. Despite its rarity, the presence of CSS signs and symptoms in a patient with a known history of HD warrants full utilization of modern diagnostic techniques. The association of ICHD with lymph node, bone, and pulmonary sites indicates that the work-up of suspected ICHD should include a minimum of a careful history and physical examination, blood and cerebrospinal fluid testing, imaging of the brain, chest, abdomen and pelvis, and bone, or PET scans [4]. To note, there is a specific subset of patients who develop painful ophthalmoplegia due to a nonspecific inflammatory process in the region of the cavernous sinus and who are exquisitely responsive to steroid treatment [8]. These patients have a disease entity which has come to be known as Tolosa–Hunt syndrome. Although the pathogenetic basis of Tolosa–Hunt syndrome remains unknown, from a practical clinical standpoint, it can be regarded as a distinct entity which may be simulated by various other disorders and is a diagnosis of exclusion after a rigorous search for other causes [8]. Had this patient not received the extensive work-up as mentioned above, she could have been misdiagnosed as having Tolosa–Hunt syndrome.

Whilst there is a paucity of literature around this topic, it must be remembered by the clinician that cavernous sinus syndrome may be the sole clinical presentation of recurrent Hodgkin's disease. Even though it is reported that lymphomatous involvement in the brain usually occurs late in the disease process, this case is evidence that CSS may precede other systemic signs and symptoms. Recognising this possibility will ensure a rigorous search for HD, an early and effective diagnosis, and a better prognosis in affected patients.

## Figures and Tables

**Table 1:** Different aetiologies of cavernous sinus syndrome.

Aetiology	Division	Example
Infection	Fungal	Mucomycosis, phycomycosis
	Viral	Herpes zoster virus opthalmicus

Tumours	Within cavernous sinus	Meningioma, schwanomma
	Compressive tumours	Pituitary adenomas, chordomas, chondrosarcomas, metastatic lesions

Vascular	Aneurysms	Cavernous segment aneurysm, intramural intracranial aneurysm
	Carotid-cavernous fistulas	Direct and indirect fistulas
	Cavernous sinus thrombosis	

Trauma		Any base of skull fracture which involves the cavernous sinus

Inflammation	Sarcoid	
	Tolosa–Hunt syndrome	

## Data Availability

The patient data used to support the findings of this study are available from the corresponding author upon request.

## Abbreviations

HD: Hodgkin's disease
CNS: Central nervous system
CSS: Cavernous sinus syndrome
ABVD: Adriamycin/ bleomycin/ vinblastine/ dacarbazine
DHAP: Dexamethasone/ high-dose aracytarabine/ platinol
OPEC: Vincristine/prednisolone/etoposide/chlorambucil
PET: Positron-emission tomography
ICHD: Intracranial Hodgkin's disease
ESR: Erythrocute sedimentation rate
HIV: Human immunodeficiency virus.
